# Dual Modification of Guar Meal via Fermentation and Enzyme Treatment Enhances Growth Performance, Nutrient Digestibility and Gut Morphology in Broilers

**DOI:** 10.1002/vms3.70785

**Published:** 2026-01-20

**Authors:** Abdul Hafeez, Wasim Akram, Shabana Naz, Rifat Ullah Khan, Ala Abudabos, Solomon Tesfay, Ibrahim A. Alhidary

**Affiliations:** ^1^ Department of Poultry Science Faculty of Animal Husbandry and Veterinary Sciences the University of Agriculture Peshawar Pakistan; ^2^ Department of Zoology Government College University Faisalabad Pakistan; ^3^ Physiology Lab, College of Veterinary Sciences Faculty of Animal Husbandry and Veterinary Sciences The University of Agriculture Peshawar Pakistan; ^4^ Department of Food and Animal Sciences College of Agriculture Tennessee State University Nashville Tennessee USA; ^5^ Department of Biology CNCS Mekelle University Mekelle Ethiopia; ^6^ Department of Animal Production College of Food and Agriculture Science King Saud University Riyadh Saudi Arabia

**Keywords:** broiler performance, enzyme treatment, fermented guar meal, intestinal histomorphology, nutrient digestibility

## Abstract

This study investigated the effects of enzyme‐treated and fermented guar meal (GM) at varying inclusion levels (3%, 6% and 9%) on broiler performance, carcass traits, nutrient digestibility and ileal histomorphology during the finisher phase. Birds fed fermented or enzyme‐treated GM at 3% and 6% levels showed significantly higher feed intake, body weight gain and improved feed conversion ratios (*p* < 0.01) compared to those receiving 9% inclusion or untreated GM. Apparent digestibility of crude protein, fibre, energy and minerals (Ca and P) was markedly enhanced in birds fed treated GM, particularly at 3% inclusion. Carcass yield and eviscerated weight were superior in fermented and enzyme‐treated groups at 3% inclusion, while abdominal fat was reduced. Histological evaluation revealed improved villus height, surface area and villus height‐to‐crypt depth ratio in treated groups, with fermented GM at 3% showing the most favourable gut morphology. In contrast, 9% untreated GM impaired nutrient utilization and gut structure. Overall, dual modification of GM via fermentation or enzyme treatment, particularly at lower inclusion levels (3% and 6%), enhances broiler performance and intestinal health, making it a viable strategy for alternative protein supplementation in broiler diets.

## Graphical abstract

Fermentation or enzyme treatment of guar meal at low inclusion levels (3%–6%) significantly improved broiler growth, nutrient digestibility, carcass yield and gut morphology, while higher inclusion impaired performance. Dual modification presents a promising strategy for safe and efficient use of guar meal as an alternative protein source in poultry diets.

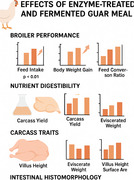

## 1 Introduction

The modern poultry industry continues to seek novel, sustainable, and cost‐effective dietary strategies to improve broiler growth efficiency while maintaining optimal health and welfare standards (Khan et al., 2023; Rahman et al. 2017; Aljohani & Zaman, 2024). Adequate provision of high‐quality protein is fundamental in poultry nutrition. Although soybean meal remains the most commonly used protein ingredient due to its favorable amino acid balance and high digestibility, escalating prices and supply constraints have encouraged interest in alternative plant‐based protein sources (Chand et al. 2018; Ullah et al. 2022; Fathanah et al., 2024; Magnoli et al., 2024). In this context, guar mealan agro‐industrial by‐product obtained during the extraction of guar gum from guar beans (Cyamopsis tetragonoloba) has emerged as a promising substitute and has attracted increasing research attention in recent years (Milczarek et al., 2022).

Guar meal possesses a favorable nutritional composition, supplying a wide range of essential amino acids such as lysine, arginine, isoleucine, tryptophan, valine, and phenylalanine. In addition, it contains comparatively higher levels of crude protein, methionine, and phosphorus than soybean meal, highlighting its potential value as a protein ingredient in poultry diets (Nidhina & Muthukumar, 2015). Despite these advantages, excessive inclusion of guar meal has been linked to negative physiological responses, including digestive disturbances, reduced growth performance, and elevated mortality. These undesirable effects are largely attributed to the presence of anti‐nutritional compounds, most notably β‐mannan, as well as saponins and trypsin inhibitors, which can impair nutrient utilization.

To mitigate these constraints, dietary enzyme supplementation has been demonstrated to enhance nutrient digestibility and alleviate the adverse effects associated with anti‐nutritional factors (Hassan et al., 2010; Nidhina & Muthukumar, 2015; Milczarek et al., 2022). Furthermore, processing approaches such as enzymatic treatment and microbial fermentation have been shown to improve the feeding value of guar meal by reducing anti‐nutrient concentrations and enhancing its suitability for inclusion in broiler diets (Abudabos et al., 2017; Milczarek et al., 2022; Boonmee et al., 2024).

Fermentation contributes to the hydrolysis of complex carbohydrate structures, thereby improving nutrient availability and minimizing the presence of deleterious compounds (Ciptaan et al., 2024; Susalam et al., 2024). In parallel, the use of exogenous enzymes promotes nutrient liberation by targeting poorly digestible fiber components, particularly galactomannans and β‐mannan. While the advantages of each strategy have been documented independently, research examining their combined application in guar meal is limited, particularly with respect to broiler growth performance and physiological adaptations during key developmental stages.

To our knowledge, this study is among the first to evaluate the combined effects of fermentation and enzyme treatment of guar meal on broiler chickens. The research aimed to assess their impact on growth performance, carcass traits, nutrient digestibility and intestinal histomorphology during the finisher phase, providing insights into the viability of this dual‐modification strategy for improving poultry feed efficiency.

## 2 Materials and Methods

### 2.1 Experimental Design, Birds and Diets

A total of 900 one‐day‐old male Hubbard broiler chicks were sourced from a commercial hatchery and used in this experiment. At placement, birds had an average initial body weight of 44.53 ± 0.15 g. Chicks were randomly allocated to nine dietary treatments in a completely randomized design, with each treatment consisting of five replicates of 20 birds each.

Throughout the starter period (days 121), all broilers received a common cornsoybean mealbased diet formulated to meet or exceed the nutritional requirements recommended for the Hubbard strain, thereby ensuring uniform nutrient intake during early growth. The experimental diets were introduced during the finisher phase (days 2242). The dietary treatments consisted of the following:

1. Control Group—Fed a basal finisher diet without any inclusion of fermented or enzyme‐treated guar meal (EGM).
2. Fermented Guar Meal (FGM) Groups—Finisher diets containing FGM at inclusion levels of 3%, 6% and 9%, labelled as FG3, FG6 and FG9.
3. EGM Groups—Finisher diets containing EGM at 3%, 6% and 9%, labelled as EG3, EG6 and EG9.

The experiment employed a 3 × 3 factorial design to evaluate both the main effects and interactions of guar meal fermentation and enzyme supplementation across graded inclusion levels (0, 3, 6, and 9%) on broiler growth performance. All finisher diets were carefully formulated to be isocaloric and isonitrogenous and met the nutritional specifications recommended for Hubbard broilers (Table 1). Feed and clean drinking water were provided ad libitum throughout the trial.

Birds were managed under standardized husbandry conditions to minimize environmental variation among treatments. Broilers were housed in environmentally controlled pens with thoroughly cleaned and disinfected floors, covered with wood‐shaving litter at a depth of approximately 57 cm. Litter was routinely turned or replaced to maintain hygienic conditions and limit ammonia accumulation. Thermal management was optimized throughout the experimental period, with brooding temperatures maintained at 3234°C during the first week and gradually reduced by 23°C per week until reaching 2426°C by day 42. A continuous lighting regimen was applied during the initial three days post‐placement to facilitate chick acclimation, followed by a lighting schedule of 16 h light and 8 h darkness for the remainder of the study.

**TABLE 1:** Feed Composition of basal diet and chemical analysis during starter phase.

Ingredients	Guar Meal		
	3%	6%	9%
Corn	67.4557	66.6272	65.7986
Soybean meal	11.2529	10.7245	10.1962
Rice polishing	9.5675	8.0028	6.4381
Guar meal	3.000	6.000	9.000
PBM/APC Hi Fat	5.000	5.000	5.000
Limestone/chips	0.8082	0.7957	0.7831
Lysine sulphate	0.8289	0.8365	0.8441
l‐VALINE	0.1902	0.1904	0.1907
l‐methionine	0.3670	0.3612	0.3554
Salt	0.1812	0.1812	0.1812
l‐Arginine	0.2767	0.2247	0.1728
Potassium carbon	0.1018	0.0839	0.0661
l‐Threonine	0.2460	0.2473	0.2487
l‐Isoleucine	0.1828	0.1812	0.1796
Sodium bicarbonate	0.1500	0.1500	0.1500
Toxin binder	0.0500	0.0500	0.0500
Vitamin premix	0.0500	0.0500	0.0500
Choline Chloride	0.1052	0.1110	0.1168
Mineral premix	0.0500	0.0500	0.0500
Betain	0.0500	0.0500	0.0500
l‐Tryptophan	0.0561	0.0523	0.0486
Anti‐oxidant	0.0100	0.0100	0.0100
Enramycin 4%	0.0100	0.0100	0.0100
Diclazuril 1%	0	0	0
Axtra PHY GOLD 1	0.0100	0.0100	0.0100
Chemical analysis			
Dry matter %	88.53	88.52	88.52
Moisture %	11.46	11.47	11.48
ME (Kcal/kg)	3050.00	3050.00	3050.00
CP %	17.64	18.47	19.29
Ash %	4.33	4.28	4.24
Fat %	5.34	5.32	5.29
Fibre %	2.93	3.00	3.07
ADF %	3.95	4.00	4.05
NDF %	10.89	10.88	10.87
Starch %	46.29	45.32	44.36
Potassium %	0.687	0.685	0.68
Calcium %	0.740	0.740	0.74

*Note*: Provided per kg of diet: vitamin A, 10,000 IU; vitamin E, 30 IU; vitamin D3, 0000 IU; thiamine, 2.5 mg; vitamin K3, 2.5 mg; pyridoxine, 5 mg; riboflavin, 5.5 mg; folic acid, 0.7 mg; vitamin B12, 0.5 mg; pantothenic acid, 15 mg; nicotinic acid, 50 mg; biotin, 0.2 mg; copper, 12 mg; manganese, 100 mg; iron, 95 mg; selenium, 0.5 mg; iodine, 0.5 mg; zinc, 100 mg.

Abbreviations: CF, crude fibre; CP, crude protein; ME, metabolizable energy.

All management, handling and sampling procedures were conducted in compliance with animal welfare guidelines to minimize stress and ensure ethical research practices.

### 2.2 Enzyme Treatment

To prepare the EGM, ground guar meal was mixed with water at a 1:1 ratio (w/v) at room temperature. The mixture was supplemented with three commercial enzyme formulations: Axta Phy (200 g/ton; DuPont, USA) containing phytase, Axtra Pro (50 g/ton; DuPont, USA) containing proteases and Hemicell (300 g/ton; Elanco, USA), a source of β‐mannanase. Additionally, an organic acid product (Silo Health, 1 kg/ton; Silo, China) was added to acidify the mixture. The entire mixture was incubated at 30°C for 24 h to facilitate enzyme activity. Following incubation, the treated material was subjected to rapid drying—completed within 3 s—to preserve nutrient integrity and inhibit microbial growth. The final product was incorporated into the finisher diets at inclusion levels of 3%, 6% and 9%, replacing a portion of the basal ration.

### 2.3 Solid‐State Fermentation of Guar Meal

Ground guar meal was thoroughly mixed with water at a 1:1 (w/v) ratio and inoculated with a commercial probiotic preparation (CLOSTAT, Kemin, USA) containing the patented Bacillus subtilis PB6 strain, following the procedure outlined by Dinani et al. (2010). The mixture was allowed to ferment for 48 h under controlled conditions, with incubation temperatures maintained between 25°C and 37°C to promote microbial activity. After fermentation, the material was oven‐dried to achieve a final moisture content of approximately 1012%, ensuring product stability and minimizing the risk of microbial spoilage during storage. The fermented guar meal was subsequently incorporated into broiler diets at graded inclusion rates of 3%, 6%, and 9% to evaluate its nutritional improvement and biological effects.

### 2.4 Evaluation of Growth Performance

Growth performance was evaluated using standard production indicators, including feed intake, body weight gain, and feed conversion ratio (FCR). Feed intake was determined as the total quantity of feed consumed per replicate throughout the experimental period and was adjusted for mortalities to accurately reflect the number of birds present at each phase. Body weight gain was calculated by subtracting initial body weight from final body weight. Feed efficiency was expressed as FCR and calculated as the ratio of mortality‐corrected feed intake to body weight gain, in accordance with the methodology described by Al‐Suwailem et al. (2024).

### 2.5 Carcass Characteristics

On Day 42 of the experiment, two birds from each replicate were randomly selected to assess carcass quality parameters. Dressed carcass weight was measured after removing feathers, blood and viscera, and expressed as a percentage of the live body weight. Eviscerated carcass weight—excluding the gastrointestinal tract, liver and other visceral organs—was also calculated and expressed similarly. The combined weights of internal organs, including the liver, heart and gizzard, were recorded in grams. Abdominal fat, comprising fat deposits around the cloaca and gizzard, was carefully excised and weighed. Meat pH was measured 24 h post‐slaughter using a calibrated digital pH meter (Hanna Instruments, USA). The probe was directly inserted into the breast muscle (pectoralis major) to obtain readings. Each sample was measured in triplicate, and the average value was recorded, following the procedure of Hafeez et al. (2025).

### 2.6 Determination of Apparent Ileal Nutrient Digestibility

On Day 38 of the trial, five birds from each replicate were randomly selected and euthanized to obtain ileal digesta samples. The ileum (portion between Meckel's diverticulum and 2 cm anterior to the ileocecal junction) was carefully excised, and the digesta were gently flushed into sterile containers. The samples were immediately chilled, freeze‐dried and ground for chemical analysis. Diets had been supplemented with chromic oxide (0.3%) as an indigestible marker to enable calculation of apparent ileal digestibility. The concentrations of dry matter (DM), CP, ether extract, crude fibre (CF) and gross energy in both diet and ileal digesta were determined following AOAC procedures (Hafeez et al. 2020).

$$\begin{array}{ccc} Nutrient\;digestibility\;\;\left(\%\right) & = & \frac{Conc.\;\;of\;marker\;in\;diet}{Conc.\;\;of\;marker\;in\;digesta}\;\\ & & \times \frac{Conc.\;\;of\;nutrient\;in\;digesta}{Conc.\;\;of\;nutrient\;in\;diet}\\ & & \times 100 \end{array}$$

### 2.7 Ileum Histomorphology

On Day 42 of the study, three birds from each treatment group were randomly selected and humanely euthanized for ileal tissue evaluation. A 2 cm segment of the ileum, positioned between Meckel's diverticulum and the ileocecal junction, was carefully excised and flushed with phosphate‐buffered saline (PBS; pH 7.4) to eliminate residual digesta, as outlined by Kalsoom (2024). The samples were fixed in 10% neutral buffered formalin for 48 h, followed by standard tissue processing, including dehydration through ascending ethanol concentrations, clearing in xylene and embedding in paraffin wax. Tissue sections approximately 4–5 µm thick were obtained using a rotary microtome and mounted onto glass slides. Slides were stained with Hematoxylin and Eosin (H&E) for microscopic examination. Histological imaging was performed using a light microscope (Olympus BX53, Japan) equipped with a digital camera and morphometric analysis was carried out using ImageJ software (NIH, USA).

### 2.8 Statistical Analysis

The experiment was conducted using a completely randomized design (CRD) with a 3 × 3 factorial arrangement to assess the effects of guar meal processing method (untreated, enzyme‐treated, and fermented) and dietary inclusion level (3%, 6%, and 9%) on growth performance and physiological responses of broilers. Statistical analyses were carried out using SPSS software (version 21.0; IBM Corp., USA). Data were analyzed by two‐way analysis of variance (ANOVA) to evaluate the main effects of processing method and inclusion level, as well as their interaction. Each replicate pen served as the experimental unit. Treatment means were considered significantly different at p < 0.05 and were compared using Tukey's multiple range test.

## 3 Results

As shown in Table 2, a significant interaction was observed between guar meal treatment types and inclusion levels on feed intake throughout the study period (*p* < 0.01**)**. In Week 4, broilers fed diets containing 3% and 6% fermented (FG3, FG6) and enzyme‐treated (EG3, EG6) guar meal exhibited significantly higher feed intake compared to those receiving 9% inclusion or untreated guar meal. This trend persisted during Weeks 5 and 6, where the highest feed intake was recorded in birds receiving FG3 and EG3, while the lowest values were consistently associated with the 9% inclusion level, particularly in the untreated (UG9) and enzyme‐treated (EG9) groups.

**TABLE 2:** Interaction effect of dietary enzyme treatment and fermentation of guar meal on Feed Intake (g) in broilers.

Weeks	UG3	UG6	UG9	EG3	EG6	EG9	FG3	FG6	FG9	SEM	*p* value
Week 4	774^ab^	762^bc^	755^c^	780^a^	774^ab^	733^d^	785^a^	779^a^	755^c^	3.14	< 0.01
Week 5	850^abc^	842^bc^	834^c^	859^ab^	851^abc^	833^c^	867^a^	858^ab^	835^c^	2.50	< 0.01
Week 6	925^b^	912^bc^	894^c^	947^a^	959^a^	913^bc^	960 ^a^	956^a^	916^b^	4.67	< 0.01
Finisher	2549^bc^	2516^cd^	2483^de^	2586^a^	2584^ab^	2479^e^	2612^a^	2593^a^	2506^de^	9.59	< 0.01
Overall	3485^c^	3422^d^	3361^e^	3541^ab^	3530^b^	3413^d^	3573^a^	3537^b^	3431^d^	13.58	< 0.01

*Note*: Different superscript letters on means in a row show significance difference at *α* = 0.05.

Abbreviations: EG3, enzyme treated guar at 3%; EG6, enzyme treated guar at 6%; EG9, enzyme treated guar at 9%; FG3, fermented guar at 3%; FG6, fermented guar at 6%; FG9, fermented guar at 9%; UG3, untreated guar at 3%; UG6, untreated guar at 6%; UG9, untreated guar at 9%.

During the finisher phase (Days 22–35), birds fed FG3 maintained the highest cumulative intake, followed closely by EG3 and FG6, while UG9 and EG9 exhibited the lowest values. The overall feed intake across the entire experimental period mirrored these findings, with fermented and EGM at 3% and 6% inclusion promoting superior feed consumption. In contrast, 9% inclusion of guar meal, regardless of processing, had a suppressive effect on feed intake.

According to Table 3, the type of guar meal treatment and inclusion level significantly influenced feed intake across all weeks and the overall study period (*p* < 0.01). FGM consistently led to higher feed intake compared to enzyme‐treated and untreated groups, with significant differences evident from Week 4 through the finisher phase. Birds fed fermented guar showed superior intake throughout, particularly during Weeks 5 and 6.

**TABLE 3:** Main treatment effect of dietary enzyme treatment and fermentation of guar meal on feed intake (g) in broilers.

	Treatments			Levels				*p* values		
Weeks	Untreated	Enzyme Treated	Fermented	3%	6%	9%	SEM	Treatment	Level	T × L
Week 4	764^b^	763^b^	773^a^	780^a^	772^b^	748^c^	3.14	< 0.01	< 0.01	< 0.01
Week 5	842^b^	848^ab^	854^a^	859^a^	850^b^	834^c^	2.50	< 0.01	< 0.01	0.23
Week 6	910^b^	939^a^	944^a^	944^a^	942^a^	908^b^	4.67	< 0.01	< 0.01	0.01
Finisher	2516^c^	2550^b^	2571^a^	2582^a^	2564^b^	2490^c^	9.59	< 0.01	< 0.01	< 0.01
Overall	3423^c^	3494^b^	3513^a^	3533^a^	3496^b^	3402^c^	13.6	< 0.01	< 0.01	< 0.01

*Note*: Mean values bearing different superscript letters in a row differ significantly (*p *< 0.05).

Among inclusion levels, 3% guar meal resulted in the highest feed intake, followed by 6%, while 9% inclusion significantly reduced feed intake (*p* < 0.01). The interaction between treatment type and inclusion level (T × L) was also significant for most weeks, suggesting that the benefits of fermentation and enzyme supplementation were most pronounced at lower inclusion rates.

As shown in Table 4, the interaction between treatment type and inclusion level of guar meal had a significant impact on broiler weight gain throughout the trial (*p* < 0.01).

**TABLE 4:** Interaction effect of dietary enzyme treatment and fermentation of guar meal on Weight Gain (g) in broilers.

	UG3	UG6	UG9	EG3	EG6	EG9	FG3	FG6	FG9	SEM	*p* value
Week 4	392^bc^	373^de^	365^e^	395^b^	392^bc^	382^cd^	398^ab^	409^a^	380^d^	2.58	< 0.01
Week 5	350^bc^	337^cd^	303^e^	363^ab^	356^ab^	335^d^	366^a^	358^ab^	329^d^	3.81	< 0.01
Week 6	362^ab^	348^bc^	328^d^	369^a^	369^a^	332^cd^	377^a^	370^a^	350^b^	3.39	< 0.01
Finisher	1104^c^	1057^d^	996^e^	1127^ab^	1116^bc^	1049^d^	1141^a^	1137^a^	1060^d^	9.17	< 0.01
Overall	1787^c^	1701^d^	1589^e^	1831^b^	1795^c^	1706^d^	1862^a^	1829^b^	1721^d^	16.0	< 0.01

*Note*: Different superscript letters on means in a row show significance difference at *α* = 0.05.

Abbreviations: EG3: Enzyme Treated Guar at 3%; EG6: Enzyme Treated Guar at 6%; EG9: Enzyme Treated Guar at 9%; FG3: Fermented Guar at 3%; FG6: Fermented Guar at 6%; FG9: Fermented Guar at 9%; UG3: Untreated Guar at 3%; UG6: Untreated Guar at 6%; UG9: Untreated Guar at 9%.

In Week 4, the highest weight gain was observed in birds fed fermented guar at 6% (FG6), followed closely by FG3 and enzyme‐treated diets at 3% and 6% (EG3, EG6). In contrast, birds receiving 9% untreated guar (UG9) showed the lowest gains, indicating a negative effect of higher inclusion levels without any processing.

During Weeks 5 and 6, birds in the fermented and enzyme‐treated groups continued to outperform those in the untreated group. Particularly, FG3 and EG3 produced significantly higher weight gains, suggesting enhanced nutrient availability with both fermentation and enzymatic treatment.

Over the finisher phase and entire trial, birds fed 3% fermented guar (FG3) achieved the greatest cumulative weight gain, followed by those in FG6 and EG3 groups. The lowest gains were consistently recorded in birds fed 9% untreated guar (UG9), reaffirming that high levels of unprocessed guar meal adversely affect growth.

The main effects of treatment type and inclusion level of guar meal on broiler weight gain were statistically significant throughout the experimental period (*p* < 0.01), as shown in Table 5.

**TABLE 5:** Main Treatment effect of dietary enzyme treatment and fermentation of guar meal on Weight Gain (g) in broilers.

	Treatments			Level				*p* values		
	Untreated	Enzyme Treated	Fermented	3%	6%	9%	SEM	Treatment	Level	T × L
Week 4	377^c^	390^b^	396^a^	395^a^	391^a^	376^b^	2.58	< 0.01	< 0.01	< 0.01
Week 5	330^b^	351^a^	351^a^	360^a^	350^b^	322^c^	3.81	< 0.01	< 0.01	0.08
Week 6	346^c^	357^b^	366^a^	369^a^	362^a^	337^b^	3.39	< 0.01	< 0.01	0.09
Finisher	1052^c^	1097^b^	1113^a^	1124^a^	1103^b^	1035^c^	9.17	< 0.01	< 0.01	< 0.01
Overall	1692^c^	1777^b^	1804^a^	1827^a^	1775^b^	1672^c^	16.0	< 0.01	< 0.01	< 0.01

*Note*: Mean values bearing different superscript letters in a row differ significantly (*p *< 0.05).

During Week 4, birds fed FGM had the highest weight gain, followed by those receiving enzyme‐treated guar. The untreated group recorded the lowest weight gain, indicating that processing techniques such as fermentation and enzymatic treatment positively influence early growth.

In Week 5, both fermented and enzyme‐treated diets supported significantly better growth compared to the untreated group. A similar trend persisted through Week 6, where the fermented group once again led in performance.

The finisher phase (Days 22–35) and the overall trial period reflected a consistent pattern: broilers consuming FGM exhibited superior weight gain, followed by those on enzyme‐treated diets. Birds fed untreated guar meal had the poorest weight performance.

Regarding inclusion levels, 3% and 6% guar meal significantly enhanced weight gain compared to 9% across all weeks. The 9% inclusion level consistently showed reduced performance, regardless of the processing method, suggesting that higher inclusion rates may impair growth even with treatment.

The FCR was significantly influenced by the interaction of treatment type and guar meal inclusion level throughout the experiment (*p* < 0.01, Table 6).

**TABLE 6:** Interaction effect of dietary enzyme treatment and fermentation of guar meal on Feed Conversion Ratio in broilers.

	UG3	UG6	UG9	EG3	EG6	EG9	FG3	FG6	FG9	SEM	*p* value
Week 4	1.97^bc^	2.05^a^	2.07^a^	1.97^bc^	1.98^b^	1.92^cd^	1.97^bc^	1.90^d^	1.98^b^	0.01	< 0.01
Week 5	2.43^bc^	2.50^bc^	2.75^a^	2.37^c^	2.39^bc^	2.49^bc^	2.37^c^	2.40^bc^	2.54^b^	0.02	< 0.01
Week 6	2.56^c^	2.62^bc^	2.73^ab^	2.57^c^	2.60^c^	2.75^a^	2.55^c^	2.58^c^	2.61^c^	0.01	< 0.01
Finisher	2.31^c^	2.38^b^	2.49^a^	2.29^c^	2.31^c^	2.37^b^	2.29^c^	2.28^c^	2.36^b^	0.01	< 0.01
Overall	1.95^de^	2.01^b^	2.12^a^	1.93^ef^	1.97^cd^	2.00^b^	1.92^f^	1.93^ef^	1.99^bc^	0.01	< 0.01

*Note*: Different superscript letters on means in a row show significance difference at *α* = 0.05.

Abbreviations: EG3, enzyme treated guar at 3%; EG6, enzyme treated guar at 6%; EG9, enzyme treated guar at 9%; FG3, fermented guar at 3%; FG6, fermented guar at 6%; FG9, fermented guar at 9%; UG3, untreated guar at 3%; UG6, untreated guar at 6%; UG9, untreated guar at 9%.

In Week 4, the best FCR was recorded in the group fed 6% FGM (FG6), followed by 9% enzyme‐treated (EG9) and 6% untreated (UG6) groups. Conversely, poorer FCR values were observed in untreated groups with higher inclusion levels, especially UG6 and UG9, indicating reduced feed efficiency.

During Week 5, the most efficient feed utilization (lowest FCR) was found in birds fed EG3, FG3 and FG6, whereas UG9 showed the highest (least favourable) FCR. This trend of improved efficiency with enzyme or fermented diets was consistent in Week 6, where the fermented and enzyme‐treated groups at lower inclusion levels (3% and 6%) performed significantly better than their untreated counterparts.

In the finisher phase (Days 22–35), broilers fed 3% and 6% levels of either enzyme‐treated or FGM showed improved FCR compared to those fed untreated diets. Among all, FG6 had the best feed efficiency.

The overall FCR across the entire study period showed that birds consuming fermented and EGM—particularly at 3% and 6% inclusion—had the most favourable conversion rates. The highest (least efficient) FCR was consistently observed in the UG9 group.

The FCR was significantly affected by both the type of guar meal treatment and the level of dietary inclusion across all experimental phases (*p* < 0.01, Table 7).

**TABLE 7:** Main Treatment effect of dietary enzyme treatment and fermentation of guar meal on FCR in broilers.

	Treatments			Level				*p* values		
	Untreated	Enzyme Treated	Fermented	3%	6%	9%	SEM	Treatment	Level	T × L
Week 4	2.03^a^	1.96^b^	1.95^b^	1.97	1.98	1.99	0.01	< 0.01	0.10	< 0.01
Week 5	2.56^a^	2.42^b^	2.44^b^	2.39 ^b^	2.43 ^b^	2.59 ^a^	0.02	< 0.01	< 0.01	0.04
Week 6	2.64^a^	2.64^a^	2.58^b^	2.56^b^	2.60^b^	2.70^a^	0.01	0.01	< 0.01	0.07
Finisher	2.39^a^	2.32^b^	2.31^b^	2.30 ^c^	2.33^b^	2.41^a^	0.01	< 0.01	< 0.01	< 0.01
Overall	2.03^a^	1.97^b^	1.95^c^	1.94^c^	1.97^b^	2.04^a^	0.01	< 0.01	< 0.01	< 0.01

*Note*: Different superscript letters on means in a row show significance difference at *α* = 0.05.

In Week 4, birds fed diets containing either enzyme‐treated or FGM exhibited better feed efficiency compared to those on untreated diets. Although the inclusion level did not show a statistically significant difference in this week (*p* = 0.10), a trend toward improved FCR with processed diets was evident.

By Week 5, both main effects (treatment and level) had a significant impact. Broilers receiving 3% and 6% inclusion levels had significantly lower FCRs compared to those fed 9%, with enzyme‐treated and fermented diets resulting in better performance than untreated ones.

During Week 6, the type of treatment again significantly influenced FCR. FGM led to improved feed utilization compared to the untreated and enzyme‐treated groups. Among inclusion levels, 3% and 6% yielded better FCRs, whereas 9% inclusion led to a decline in efficiency.

In the finisher phase (Day 22–35), birds fed fermented, or EGM, performed significantly better than those on untreated diets. The 3% inclusion level resulted in the best FCR, while 9% had the least favourable outcome, likely due to ANFs in higher concentrations.

A significant interaction (*p* < 0.01) was observed between processing method (untreated, enzyme‐treated, fermented) and inclusion level (3%, 6% and 9%) for both dressing percentage and eviscerated carcass weight (Table 8).

**TABLE 8:** Interaction effect of dietary enzyme treatment and fermentation of guar meal on carcass characteristics in broilers.

Groups	UG3	UG6	UG9	EG3	EG6	EG9	FG3	FG6	FG9	SEM	*p* value
Dressing %	67.4^a^	63.8^b^	61.0^c^	68.0^a^	67.1^a^	64.5^b^	67.9^a^	67.3^a^	64.2^b^	0.45	< 0.01
Eviscerated weight (%)	76.4^ab^	70.7^c^	70.5^c^	78.0^ab^	77.7^ab^	74.0^bc^	79.5^a^	79.4^a^	75.6^abc^	0.69	< 0.01
Giblet weight (g)	75.0	86.0	75.3	81.7	74.3	74.7	76.7	75.7	77.0	1.09	0.14
Abdominal fat weight (g)	1.69^ab^	1.86^a^	1.87^a^	1.75^ab^	1.68^ab^	1.80^ab^	1.63^b^	1.66^ab^	1.86^a^	0.02	< 0.01
Meat pH	5.73	5.73	5.67	5.70	5.73	5.73	5.67	5.60	5.70	0.04	1.00
Meat colour index	1.67	1.67	1.67	1.33	1.33	1.33	1.67	1.67	1.67	0.10	0.97

*Note*: Different superscript letters on means in a row show significance difference at *α* = 0.05.

Abbreviations: EG3, enzyme treated guar at 3%; EG6, enzyme treated guar at 6%; EG9, enzyme treated guar at 9%; FG3, fermented guar at 3%; FG6, fermented guar at 6%; FG9, fermented guar at 9%; UG3, untreated guar at 3%; UG6, untreated guar at 6%; UG9, untreated guar at 9%.

The highest dressing percentages were recorded in broilers fed diets containing 3% guar meal, regardless of processing method (UG3, EG3 and FG3), indicating superior carcass yield at lower inclusion levels. Notably, the lowest dressing percentage was observed in the untreated 9% group (UG9).

Similarly, eviscerated weight percentages were significantly enhanced in birds fed fermented and enzyme‐treated diets at lower inclusion levels (FG3, FG6, EG3 and EG6), with values exceeding 77%, whereas the lowest values were again recorded in UG6 and UG9 groups, suggesting a detrimental impact of higher untreated guar meal levels.

For abdominal fat weight, significant differences (*p* < 0.01) were also noted. Birds fed FG3 exhibited the lowest fat deposition, while higher values were found in UG6, UG9 and FG9, indicating that fermentation may help reduce fat accumulation, especially at moderate inclusion levels.

There were no significant differences observed for giblet weight, meat pH, or meat colour index across treatments (*p* > 0.05), suggesting that neither guar processing method nor inclusion level influenced these traits.

A significant effect of guar meal processing method and inclusion level was observed on dressing percentage and eviscerated carcass yield (*p* < 0.01) as shown in Table 9. Birds fed enzyme‐treated and FGM diets had higher dressing percentages (∼66.5%) compared to those receiving untreated guar meal (64.1%). The highest carcass yields were recorded at the 3% inclusion level, while a decline in performance was noted at 9% inclusion, regardless of treatment.

**TABLE 9:** Main Treatment effect of dietary enzyme treatment and fermentation of guar meal on Carcass characteristics in broilers.

Treatment	Treatment			Level				*p* values		
Level	Untreated	Enzyme Treated	Fermented	3%	6%	9%	SEM	Treatment	Level	T × L
Dressing %	64.1^b^	66.5^a^	66.5^a^	67.7^a^	66.1^b^	63.3^c^	0.45	0.00	< 0.01	< 0.01
Eviscerated weight (%)	72.5^b^	76.6^a^	78.2^a^	77.9^a^	75.9^a^	73.3^b^	0.69	0.00	< 0.01	0.09
Giblet weight (g)	78.8	76.9	76.4	77.8	78.7	75.7	1.09	0.59	0.45	0.05
Abdominal Fat weight (g)	1.81	1.74	1.72	1.69^b^	1.74^b^	1.84^a^	0.02	0.07	< 0.01	0.07
Meat pH	5.71	5.72	5.66	5.70	5.69	5.70	0.04	0.81	0.99	0.97
Meat colour index	1.67	1.33	1.67	1.56	1.56	1.56	0.10	0.39	1.00	1.00

*Note*: Means in the same row with different superscript letters are significantly different at *α* = 0.05.

Likewise, eviscerated weight percentage was significantly influenced by treatment. Both fermented and enzyme‐treated groups showed superior yields (∼76.6%–78.2%), significantly outperforming the untreated group (72.5%). Birds fed 3% and 6% guar meal exhibited better performance than those fed at 9%.

In contrast, giblet weights and meat pH remained statistically unaffected by either treatment or inclusion level (*p* > 0.05), indicating no notable influence on these parameters.

A trend toward reduced abdominal fat deposition was observed in birds receiving lower levels (3% and 6%) of guar meal, especially in fermented groups. However, the overall treatment effect on fat weight was not statistically significant (*p* = 0.07), though the inclusion level was (*p* < 0.01).

The meat colour index showed no significant variation among treatments or inclusion levels.

The interaction between dietary treatments and inclusion levels significantly influenced the digestibility of CP, CF, nitrogen‐free extract (NFE), calcium (Ca) and phosphorus (P) (*p* < 0.05), while the digestibility of ash remained statistically unaffected (*p* = 0.40). DM digestibility showed a borderline significance (*p* = 0.05; Table 10).

**TABLE 10:** Interaction effect of dietary enzyme treatment and fermentation of guar meal on percent apparent total digestibility (ATD) of nutrients at finisher phase in broilers.

Groups	UG3	UG6	UG9	EG3	EG6	EG9	FG3	FG6	FG9	SEM	*p* value
DM	71.4	72.0	74.3	73.4	73.1	73.9	75.5	72.8	75.2	0.35	0.05
ASH	44.5	46.4	45.1	47.9	47.0	46.7	47.0	45.8	45.2	0.36	0.40
CP	65.6^bc^	62.3^cde^	57.8^e^	71.9^a^	68.6^ab^	60.2^de^	73.0^a^	71.7^a^	63.3^cd^	0.93	< 0.01
CF	77.1^a^	70.6^bc^	65.4^c^	77.8^a^	74.7^ab^	70.1^bc^	77.7^a^	77.1^a^	72.5^ab^	0.77	< 0.01
NFE	81.1^ab^	77.9^cd^	77.3^d^	82.9^a^	81.1^ab^	80.3^bc^	83.5^a^	83.7^a^	78.8^bcd^	0.41	< 0.01
Ca	26.0^ab^	26.0^ab^	23.4^b^	27.7^a^	26.0^ab^	25.3^ab^	27.6^a^	26.8^a^	25.8^ab^	0.29	0.01
P	27.7^a^	25.6^bc^	23.4^d^	28.9^a^	27.1^ab^	24.8^cd^	29.0^a^	27.2^ab^	25.6^bc^	0.32	< 0.01

*Note*: Different superscript letters on means in a row show significance difference at *α* = 0.05.

Abbreviations: EG3, enzyme treated guar at 3%; EG6, enzyme treated guar at 6%; EG9, enzyme treated guar at 9%; FG3, fermented guar at 3%; FG6, fermented guar at 6%; FG9, fermented guar at 9%; UG3, untreated guar at 3%; UG6, untreated guar at 6%; UG9, untreated guar at 9%.

CP digestibility was highest in broilers fed FGM at 3% and 6%, as well as enzyme‐treated guar at 3%, indicating improved protein utilization. The lowest CP digestibility was recorded in the group receiving untreated guar meal at 9%, highlighting the negative impact of unprocessed guar meal at higher inclusion levels.

CF digestibility also benefited from fermentation and enzyme treatments. The highest values were observed in all groups with 3% inclusion of treated guar meal (both FG and EG), whereas untreated diets, especially at 9%, resulted in reduced fibre digestibility.

For NFE, digestibility was significantly enhanced in the fermented and enzyme‐treated groups, with FG3 and FG6 showing superior values. In contrast, UG9 (untreated at 9%) exhibited the lowest digestibility, again underscoring the limitations of high‐level unprocessed inclusion.

Calcium (Ca) and phosphorus (P) digestibility was significantly improved in enzyme‐treated and fermented groups at 3%, with EG3 and FG3 showing the best results for both minerals. Notably, P digestibility dropped markedly in the untreated groups, particularly at 9% inclusion, suggesting impaired mineral availability. Ash digestibility, though numerically higher in enzyme‐treated groups, did not differ significantly among treatments.

The main effects of dietary treatments (untreated, enzyme‐treated and FGM) and inclusion levels (3%, 6% and 9%) significantly influenced the apparent total digestibility (ATD) of several nutrients in broilers during the finisher phase (Table 11).

**TABLE 11:** Main Treatment effect of dietary enzyme treatment and fermentation of guar meal on percent apparent total digestibility (ATD) of nutrients at finisher phase in broilers.

	Treatment			Level				*p* values		
	Untreated	Enzyme Treated	Fermented	3%	6%	9%	SEM	Treatment	Level	T × L
DM	72.6^b^	73.5^ab^	74.5^a^	73.4^ab^	72.6^b^	74.5^a^	0.35	0.05	0.06	0.28
ASH	45.3	47.2	46.0	46.5	46.4	45.6	0.36	0.11	0.57	0.59
CP	61.9^c^	66.9^b^	69.3^a^	70.2^a^	67.5^b^	60.4^c^	0.93	< 0.01	< 0.01	0.16
CF	71.0^b^	74.2^a^	75.8^a^	77.5^a^	74.1^b^	69.4^c^	0.77	< 0.01	< 0.01	0.08
NFE	78.7^b^	81.4^a^	82.0^a^	82.5 ^a^	80.9^b^	78.8^c^	0.41	< 0.01	< 0.01	< 0.01
Ca	25.1^b^	26.3^ab^	26.7^a^	27.1 ^a^	26.3^a^	24.8^b^	0.29	0.03	< 0.01	0.62
P	25.6^b^	26.9^a^	27.2^a^	28.5^a^	26.6^b^	24.6^c^	0.32	< 0.01	< 0.01	0.81

*Note*: Means in the same row with different superscript letters are significantly different at *α* = 0.05.

DM digestibility was significantly higher in the fermented group compared to the untreated, with enzyme‐treated birds showing intermediate values (*p* = 0.05). Among inclusion levels, 9% yielded the highest DM digestibility, while 6% showed the lowest (*p* = 0.06).

CP digestibility improved markedly in the enzyme‐treated and fermented groups (*p* < 0.01), with the highest value observed at 3% inclusion, and the lowest at 9%. This indicates that both enzyme and fermentation treatments enhance protein utilization, particularly at lower levels.

CF digestibility was also significantly affected by treatment and inclusion level (*p* < 0.01). Fermented and enzyme‐treated diets resulted in better fibre digestibility, with a peak at 3% inclusion, suggesting improved breakdown of complex fibres by microbial and enzymatic activity.

NFE digestibility showed a strong treatment and level effect (*p* < 0.01). The fermented diets, especially at 3%, achieved the highest digestibility values, whereas untreated and higher inclusion diets showed reduced efficiency.

Calcium (Ca) and Phosphorus (P) digestibility were both enhanced by fermentation and enzyme supplementation (*p* = 0.03 and *p* < 0.01, respectively). The highest mineral digestibility was recorded at 3% inclusion, confirming that both treatments enhance mineral bioavailability, likely due to phytate degradation and reduced anti‐nutritional interference.

The interaction between dietary treatments and inclusion levels had a statistically significant impact on all evaluated ileal histomorphological parameters, except for lamina propria thickness (*p* = 0.15), as presented in Table 12.

**TABLE 12:** Interaction effect of dietary enzyme treatment and fermentation of guar meal on histomorphology of ileum in broilers.

Groups	UG3	UG6	UG9	EG3	EG6	EG9	FG3	FG6	FG9	SEM	*p* value
Villus length (mm)	1.37^c^	1.34^c^	0.97^d^	1.92^b^	1.70^b^	1.26^c^	2.34^a^	1.69^b^	1.26^c^	0.06	< 0.01
Villus width (mm)	0.10^bc^	0.09^c^	0.09^c^	0.12^ab^	0.12^ab^	0.10^bc^	0.13^a^	0.12^ab^	0.10^bc^	< 0.01	< 0.01
Crypt depth (mm)	0.25^b^	0.32^ab^	0.40^a^	0.25^ab^	0.31^ab^	0.33^ab^	0.33^ab^	0.25^b^	0.27^ab^	0.01	0.03
Lamina propria (µm)	0.06	0.06	0.06	0.04	0.06	0.07	0.05	0.04	0.06	< 0.01	0.15
V length: C depth	5.88^abc^	4.30^cd^	2.96^d^	8.06^a^	5.81^abc^	3.94^cd^	7.25^a^	7.04^ab^	4.64^bcd^	0.28	< 0.01
Villus surface area (mm^2^)	0.41^c^	0.38^cd^	0.28^d^	0.74^b^	0.64^b^	0.38^cd^	0.93^a^	0.63^b^	0.40^c^	0.03	< 0.01

*Note*: Different superscript letters on means in a row show significance difference at *α* = 0.05.

Abbreviations: EG3, enzyme treated guar at 3%; EG6, enzyme treated guar at 6%; EG9, enzyme treated guar at 9%; FG3, fermented guar at 3%; FG6, fermented guar at 6%; FG9, fermented guar at 9%; UG3, untreated guar at 3%; UG6, untreated guar at 6%; UG9, untreated guar at 9%.

Villus length was notably affected by both treatment type and inclusion level. The greatest villus height was observed in broilers fed FGM at 3% (FG3), reaching 2.34 mm, followed by EG3 (1.92 mm) and FG6 (1.69 mm). In contrast, the shortest villi were recorded in the UG9 group (0.97 mm), demonstrating the adverse effects of high levels of unprocessed guar meal on intestinal structure.

Similarly, villus width was significantly influenced by the treatments, with the widest villi found in FG3 (0.13 mm). The narrowest villi were seen in UG6 and UG9 (0.09 mm). Both enzyme treatment and fermentation improved villus thickness compared to the untreated control groups, indicating enhanced mucosal development and absorptive surface.

Crypt depth, another key indicator of intestinal health, varied across treatments. The deepest crypts were observed in UG9 (0.40 mm), which may suggest a compensatory response to epithelial damage or increased cellular turnover. In contrast, more favourable values were recorded in FG3 and EG3 (∼0.25 mm), indicating balanced crypt architecture and healthier intestinal lining.

The villus height to crypt depth ratio (V:C)—a sensitive marker of gut functionality—was significantly highest in EG3 (8.06) and FG3 (7.25), reflecting superior absorptive potential and epithelial renewal rates. Conversely, the lowest V:C ratio occurred in UG9 (2.96), again highlighting the detrimental effects of higher inclusion of untreated guar meal.

In terms of villus surface area, which is critical for nutrient absorption, FG3 achieved the largest value (0.93 mm^2^), followed by EG3 (0.74 mm^2^). The smallest surface area was associated with UG9 (0.28 mm^2^). These findings confirm that both fermentation and enzymatic treatment at lower inclusion levels greatly enhance gut morphology, which is essential for optimal nutrient assimilation in broilers.

As shown in Table 13, notable differences in ileal histomorphological parameters were observed in response to the type of guar meal processing (untreated, enzyme‐treated, or fermented) and its inclusion level in broiler diets.

**TABLE 13:** Main Treatment effect of dietary enzyme treatment and fermentation on histomorphology of ileum in broilers.

Treatment	Treatment			Level				*p* values		
Level	Untreated	Enzyme Treated	Fermented	3%	6%	9%	SEM	Treatment	Level	T × L
Villus length (mm)	1.22^c^	1.62^b^	1.76^a^	1.88^a^	1.58^b^	1.16^c^	0.06	< 0.01	< 0.01	< 0.01
Villus width (mm)	0.09^b^	0.11^a^	0.12^a^	0.12^a^	0.11^a^	0.10^b^	0.00	< 0.01	< 0.01	0.16
Crypt depth (mm)	0.32	0.30	0.28	0.27	0.29	0.33	0.01	0.32	0.09	0.03
Lamina propria (µm)	0.06	0.06	0.05	0.05	0.05	0.06	0.00	0.23	0.12	0.27
V length:C depth	4.38^b^	5.94^a^	6.31^a^	7.06^a^	5.72^b^	3.85^c^	0.28	< 0.01	< 0.01	0.39
Villus surface area (mm^2^)	0.36^c^	0.59^b^	0.65^a^	0.69^a^	0.55^b^	0.35^c^	0.03	< 0.01	< 0.01	< 0.01

*Note*: Means in the same row with different superscript letters are significantly different at *α* = 0.05.

Villus length was significantly influenced by both dietary treatments and inclusion levels (*p* < 0.01). Broilers receiving FGM exhibited the longest villi (1.76 mm), followed closely by those on enzyme‐treated guar diets (1.62 mm). In contrast, the shortest villi (1.22 mm) were seen in birds fed untreated guar meal. Inclusion level also played a critical role, with the greatest villus height recorded at 3% (1.88 mm), while a marked reduction was noted at the 9% inclusion level (1.16 mm), indicating potential mucosal stress or compromised gut development at higher concentrations.

Villus width followed a similar trend, with significantly broader villi in enzyme‐treated (0.11 mm) and fermented groups (0.12 mm) than in the untreated group (0.09 mm) (*p* < 0.01). However, variation across inclusion levels was minimal, suggesting the processing method had a more dominant influence than the inclusion rate.

Crypt depth did not show significant differences among the treatment groups (*p* = 0.32), though a significant interaction effect (*p* = 0.03) indicates that the combination of treatment and inclusion level may influence this parameter under certain conditions. This suggests that while individual treatments may not alter crypt depth markedly, their interaction with inclusion level might impact epithelial regeneration.

Lamina propria thickness remained unaffected across all treatments and inclusion levels (*p* > 0.05), implying that submucosal integrity was preserved regardless of dietary intervention.

The villus height to crypt depth ratio (V:C), a crucial indicator of intestinal efficiency and epithelial turnover, was significantly enhanced in broilers fed enzyme‐treated (5.94) and FGM (6.31) diets (*p* < 0.01). The highest V:C ratio was associated with the 3% inclusion level (7.06), whereas the lowest was found at 9% inclusion (3.85), pointing to a decline in gut efficiency at higher inclusion rates.

Lastly, villus surface area, an important measure of nutrient absorptive potential, was significantly greater in birds fed fermented (0.65 mm^2^) and enzyme‐treated guar (0.59 mm^2^) compared to those fed untreated guar (0.36 mm^2^). Inclusion levels followed a similar pattern, with the highest surface area observed at 3% (0.69 mm^2^) and the lowest at 9% (0.35 mm^2^) (*p* < 0.01), reinforcing the beneficial effects of both processing and moderation in inclusion rates for optimal gut morphology.

## 4 Discussion

Recent research highlights the importance of innovative nutritional and management strategies, including feed additives, protein optimization, phytobiotics and probiotics, in enhancing poultry growth, health and overall production efficiency (Chand et al. 2018; Habib et al. 2024; Dinasarki et al. 2024; Othman et al. 2024; Usman et al. 2024). The present study revealed that dietary inclusion of GM, when subjected to enzyme supplementation or microbial fermentation, significantly enhanced FI, WG and FCR in broilers. These improvements were influenced by both processing method and inclusion level, with notable interaction effects observed. Feed intake peaked in birds receiving 3% and 6% fermented or enzyme‐treated GM (particularly FG3 and EG3), while the lowest intake was recorded in the 9% untreated and enzyme‐treated groups (UG9 and EG9). This trend suggests that both fermentation and enzymatic treatment mitigate the negative effects of ANFs in GM, likely through improved palatability and nutrient accessibility—findings that align with those of Woyengo et al. (2022), Jabbar, Tahir, Alhidary, et al. (2021), Jabbar, Tahir, Khan, et al. (2021), and Xu et al. (2012).

Notably, the highest WG occurred in broilers fed fermented GM at 3% and 6% (FG3 and FG6). These outcomes are consistent with studies by Elbaz et al. (2021) and Akram et al. (2025), which highlighted the ability of fermentation to enhance nutrient availability through microbial breakdown of complex polysaccharides and production of organic acids. While enzyme‐treated GM also improved WG compared to untreated GM, its effects were slightly less pronounced than fermentation. The poorest WG and FCR were observed in the UG9 group, underscoring the negative impact of higher GM inclusion without processing, likely due to unresolved ANFs and decreased digestibility (Toghyani et al. 2017; Hafeez et al. 2025).

The FCR data mirrored the patterns observed in FI and WG, with the lowest (best) FCR values recorded in the FG3 and FG6 groups, followed by EG3. These improvements in feed efficiency are attributed to reduced gut viscosity, enhanced protein and energy digestibility and better intestinal health—benefits also reported by Ahsan et al. (2024). However, at the 9% inclusion level, even processed GM could not fully offset the negative effects, potentially due to nutrient dilution, palatability issues, or suboptimal fermentation—concerns echoed in over‐inclusion studies by Elbaz et al. (2023) and Toghyani et al. (2017).

Collectively, these findings highlight the complementary mechanisms of fermentation and enzyme supplementation in enhancing GM utilization in broilers. Enzymes such as proteases, phytases and β‐mannanases degrade non‐starch polysaccharides like galactomannans, improving nutrient digestibility and reducing intestinal viscosity (Hafeez et al. 2025). Meanwhile, microbial fermentation not only reduces ANFs but also introduces bioactive metabolites and organic acids that promote gut health and nutrient absorption (Jabbar, Tahir, Alhidary, et al. 2021; Jabbar, Tahir, Khan, et al. 2021; Xu et al. 2012). This dual strategy effectively transforms GM—a low‐cost alternative protein source—into a more functional and digestible feed ingredient, supporting sustainable and performance‐driven broiler nutrition.

Broilers fed 3% guar meal—especially fermented or enzyme‐treated (FG3, EG3)—showed the highest dressing percentages and carcass weights, while the 9% untreated group (UG9) had the lowest, indicating that low‐level processed GM enhances yield, whereas higher unprocessed GM impairs carcass traits. This finding aligns with Toghyani et al. (2017), who demonstrated that enzyme supplementation in canola‐based diets improved nutrient absorption, leading to enhanced carcass traits. The main effects further confirm that enzyme and fermentation treatments significantly improved eviscerated yield (∼76.6–78.2%), outperforming the untreated group (72.5%).

The lowest fat weights were recorded in broilers fed FG3, suggesting that moderate fermentation promotes lean tissue accretion and limits lipid storage. In contrast, higher abdominal fat was noted in UG6, UG9 and FG9. These trends agree with the findings of Xu et al. (2012), Jabbar, Tahir, Alhidary, et al. (2021), Jabbar, Tahir, Khan, et al. (2021) and Ahsan et al. (2024). Hafeez et al. (2025), who reported that excessive inclusion of fibrous or fermented ingredients may alter lipid metabolism, potentially resulting in higher fat accumulation. Thus, moderate processing combined with optimal inclusion levels is essential to realize the full potential of guar meal in broiler nutrition. Excessive inclusion, even with fermentation or enzymes, may lead to diminishing returns or metabolic burdens, echoing conclusions from studies on other high‐fibre feed ingredients in poultry diets (Elbaz et al. 2021, 2023; Hafeez et al. 2025).

The present study demonstrated that enzyme supplementation and fermentation of GM significantly improved the ATD of key nutrients—particularly CP, CF, NFE, Ca and P—in broilers. Although DM digestibility showed only marginal statistical significance, a clear trend was observed in which birds fed fermented and enzyme‐treated GM, especially at 3% inclusion, exhibited numerically higher digestibility values than those fed untreated GM. This aligns with earlier findings by Woyengo et al. (2022) and Ahsan et al. (2024), who reported that enzyme supplementation enhances digestibility by degrading ANFs and complex polysaccharides. The borderline significance in DM digestibility in this study may be due to the limited digestibility improvement for structural components such as cellulose, which are less influenced by these treatments alone (Hafeez et al. 2025).

The digestibility of CP improved significantly with both enzyme‐treated and fermented GM, particularly at 3% inclusion. Birds fed FG3, FG6 and EG3 recorded the highest CP digestibility, indicating enhanced protein utilization through microbial proteolysis or enzymatic hydrolysis. These results are in line with the work of Jabbar, Tahir, Alhidary, et al. (2021) and Jabbar, Tahir, Khan, et al. (2021), who emphasized the potential of fermentation and exogenous proteases to increase protein bioavailability in plant‐based feed ingredients. Similarly, CF digestibility was highest in birds fed 3% fermented or enzyme‐treated GM, highlighting the ability of microbial fermentation to degrade structural carbohydrates via enzymes such as cellulases and hemicellulases (Hafeez et al. 2025). While exogenous enzymes also improved CF digestibility, fermentation consistently outperformed enzyme‐only treatments, suggesting a broader enzymatic spectrum generated during microbial fermentation (Ahsan et al. 2024).

The digestibility of NFE was significantly enhanced by both treatments, with the highest values in FG3 and FG6. This indicates improved carbohydrate digestion and utilization, potentially due to the degradation of soluble NSPs and starch components during fermentation (Hafeez et al. 2025). Interestingly, NFE digestibility was also significantly influenced by inclusion level, with the lowest values in UG9, reflecting how high levels of untreated GM impair carbohydrate absorption, likely through increased gut viscosity. In terms of mineral digestibility, Ca and P availability improved significantly in response to fermentation and enzyme treatment. The highest values were observed in FG3 and EG3, suggesting enhanced mineral solubility and reduced interference from phytates or tannins. These findings echo the conclusions of Ahsan et al. (2024) and Hafeez et al. (2025), who demonstrated that fermentation reduces phytate content, thus improving mineral bioavailability. Additionally, enzyme supplementation may indirectly support Ca and P digestibility by reducing dietary fibre complexity and enhancing the overall gut environment.

However, P digestibility, despite being significantly influenced by treatment and inclusion level, may have shown less pronounced improvements than expected due to the absence of phytase in the diets. Phytase is specifically required to hydrolyse phytate‐bound phosphorus, and its exclusion could explain why phosphorus digestibility, although improved, did not reach maximal levels (Woyengo et al. 2022; Jabbar, Tahir, Alhidary, et al. 2021; Jabbar, Tahir, Khan, et al. 2021).

The present study demonstrated that the most notable improvement in villus length and width was observed in birds fed fermented (FG3, FG6) and enzyme‐treated diets (EG3, EG6), particularly at the 3% inclusion level. These enhancements highlight the detrimental effects of high levels of unprocessed GM and confirm the histological benefits of fermentation and enzyme application. Similar trends were reported by Hafeez et al. (2025), who showed that fermented GM improved nutrient digestibility via enhanced villus structure, and by Afzal et al. (2016), who found that prebiotics and enzymes support villus growth and mucosal health.

Enzyme supplementation, especially with β‐mannanase, likely contributed to the breakdown of viscous NSPs such as galactomannans, improving nutrient diffusion and reducing mucosal irritation, which supports wider villi observed in EG3 (Abd El‐Wahab et al. 2022; Z. F. Zhang and Kim 2013). These effects parallel those observed by A. W. Zhang et al. (2005), who demonstrated that protease supplementation increases villus surface area and nutrient absorptive capacity. Crypt depth was greatest in UG9, indicating intestinal stress and elevated epithelial turnover due to unresolved ANFs. In contrast, more moderate crypt depths in FG3 and EG3 reflected healthier mucosal structure, aligning with findings by and Liu et al. (2021), who reported reduced crypt hyperplasia following fermentation and enzyme application. Although treatment had no significant main effect on crypt depth, a significant interaction suggests that crypt morphology was influenced by both inclusion level and processing method, consistent with Long et al. (2021).

The villus height‐to‐crypt depth (V:C) ratio—a critical indicator of absorptive efficiency—was significantly improved in EG3 and FG3, reflecting enhanced intestinal function and epithelial stability. This corroborates previous studies by Woyengo et al. (2022) and Z. F. Zhang and Kim (2013), all of which associated improved V:C ratios with enzyme and fermentation‐based dietary interventions. Villus surface area, another key determinant of nutrient absorption, followed a similar pattern: FG3 and EG3 groups recorded the highest values, supporting observations by Akram et al. (2025) and A. W. Zhang et al. (2005), who attributed increased surface area to microbial stimulation of enterocyte proliferation and microvilli expansion. These findings underscore the importance of processing in maximizing gut absorptive capacity, particularly at moderate inclusion levels.

It should be acknowledged that this study employed only a single microbial strain (Saccharomyces cerevisiae) with β‐mannanase under a fixed 48 h solid‐state fermentation protocol, and digestibility was assessed using short‐term collection. While the results were promising, responses may differ with other microbial–enzyme combinations, fermentation conditions, or over longer‐term adaptation, warranting further investigation.

## 5 Conclusion

In conclusion, enzyme and fermentation processing of guar meal significantly enhanced feed intake, weight gain, feed efficiency, nutrient digestibility, carcass yield and gut morphology in broilers, particularly at 3% and 6% inclusion levels. However, higher inclusion rates (9%) of untreated guar meal consistently impaired performance and intestinal health, highlighting the importance of both processing method and optimal inclusion level.

## Author Contributions

Conceptualisation: Abdul Hafeez. Methodology: Wasim Akram. Validation: Rifat Ullah Khan and Solomon Tesfay. Writing – original draft review and editing: Shabana Naz, Ala Abudabos and Ibrahim A. Alhidary. All authors have read and agreed to the published version of the manuscript.

## Funding

The authors have nothing to report.

## Ethics Statement

The study was approved by the Ethical Committee of the Faculty of Animal Husbandry & Veterinary Sciences, The University of Agriculture, Peshawar, Pakistan (Approval No. 12/FAH&VS/2021).

## Conflicts of Interest

The authors declare no conflicts of interest.

## Data Availability

The relevant data are provided in the paper. The data of the current experiment can be obtained from the corresponding author when needed.
